# Soft three-dimensional network materials with rational bio-mimetic designs

**DOI:** 10.1038/s41467-020-14996-5

**Published:** 2020-03-04

**Authors:** Dongjia Yan, Jiahui Chang, Hang Zhang, Jianxing Liu, Honglie Song, Zhaoguo Xue, Fan Zhang, Yihui Zhang

**Affiliations:** 1 Applied Mechanics Laboratory, Department of Engineering Mechanics, Tsinghua University, Beijing, 100084 People’s Republic of China; 2 Center for Flexible Electronics Technology, Tsinghua University, Beijing, 100084 People’s Republic of China

**Keywords:** Mechanical engineering, Materials for devices, Soft materials

## Abstract

Many biological tissues offer J-shaped stress–strain responses, since their microstructures exhibit a three-dimensional (3D) network construction of curvy filamentary structures that lead to a bending-to-stretching transition of the deformation mode under an external tension. The development of artificial 3D soft materials and device systems that can reproduce the nonlinear, anisotropic mechanical properties of biological tissues remains challenging. Here we report a class of soft 3D network materials that can offer defect-insensitive, nonlinear mechanical responses closely matched with those of biological tissues. This material system exploits a lattice configuration with different 3D topologies, where 3D helical microstructures that connect the lattice nodes serve as building blocks of the network. By tailoring geometries of helical microstructures or lattice topologies, a wide range of desired anisotropic J-shaped stress–strain curves can be achieved. Demonstrative applications of the developed conducting 3D network materials with bio-mimetic mechanical properties suggest potential uses in flexible bio-integrated devices.

## Abstract (web summary)

The development of artificial 3D soft materials and device systems that can reproduce the nonlinear, anisotropic mechanical properties of biological tissues remains challenging. Here, the authors design a class of soft 3D network materials that can offer defect-insensitive, nonlinear mechanical responses closely matched with those of biological tissues.

## Introduction

Inspired by the microstructure constructions of biological tissues, a number of artificial soft materials have been developed recently to offer similar, bio-mimetic physical properties^1–13^. These artificially engineered materials hold promising applications in tissue engineering^14–17^, soft robotics^18–26^, biomedical devices^27–37^, and other areas^38–41^. Existing studies show that most biological tissues, such as skin^42,43^, ligaments^44^, cardiac tissue^45^, and blood vessel^46^, are comprised mainly of curved and chained microstructures (e.g., collagen triple helix, collagen fibril, collagen fiber, and etc.). Upon uniaxial stretching, those microstructures in biological tissues unravel to align with the loading direction at the initial stage of stretching, and begin to straighten once the microstructures are fully extended at a relatively large level of stretching. Such a deformation mechanism gives rise to a “J-shaped” stress–strain response, in which the tangent modulus typically increases with increasing the applied strain, due to the transition of bending-dominated mode to stretching-dominated mode. For two-dimensional (2D) biological tissues (e.g., skin^47^), a soft network design that incorporates horseshoe microstructures into periodic lattice constructions has been developed, which can be tailored precisely to match the J-shaped stress–strain curves of human skins at diverse locations^48,49^. However, this type of 2D network design cannot be extended directly to three-dimensional (3D) cases, due to the 2D nature of horseshoe microstructures. Although some previous studies^50–54^ reported synthetic materials that can reproduce the linear mechanical properties of biological tissues at small strains, it is still very challenging to develop soft 3D architected materials that can mimic the nonlinear, anisotropic mechanical responses of 3D biological tissues.

Many collagenous tissues are found to possess a type of helix-shaped 3D microstructures^2,43,55,56^, some of which exhibit regular geometric configurations. These helix-shaped 3D microstructures are crucial to the J-shaped stress–strain responses^57–60^. Inspired by this type of 3D helical microstructures, we introduce a bio-mimetic design of soft 3D network materials based on periodic 3D lattice configurations, in which periodically arranged helical microstructures serve as building blocks that connect the lattice nodes. Different from metal/polymer foams that render J-shaped stress–strain curves only under compression^61–63^, the soft 3D network materials developed herein offer J-shaped stress–strain responses under both the compression and stretching, almost along an arbitrary loading direction. For random defects in the form of missing microstructures, the experimental measurements suggest a defect-insensitive behavior of soft 3D network materials with defect densities up to 5%. Quantitative mechanics modeling well captures the effects of key design parameters on the nonlinear mechanical responses, which provides a design tool to achieve desired isotropic/anisotropic stress–strain curves of real biological tissues (e.g., heart muscles), by tuning the geometry of helical microstructures. Integration of conducting layers with soft 3D network materials allows the development of flexible pressure sensors and stretchable conductors with J-shaped stress–strain curves matched with that of biological tissues, indicating the potential applications in biomedical devices.

## Results

### Bio-mimetic design and fabrication of network materials

**Fig. 1:**
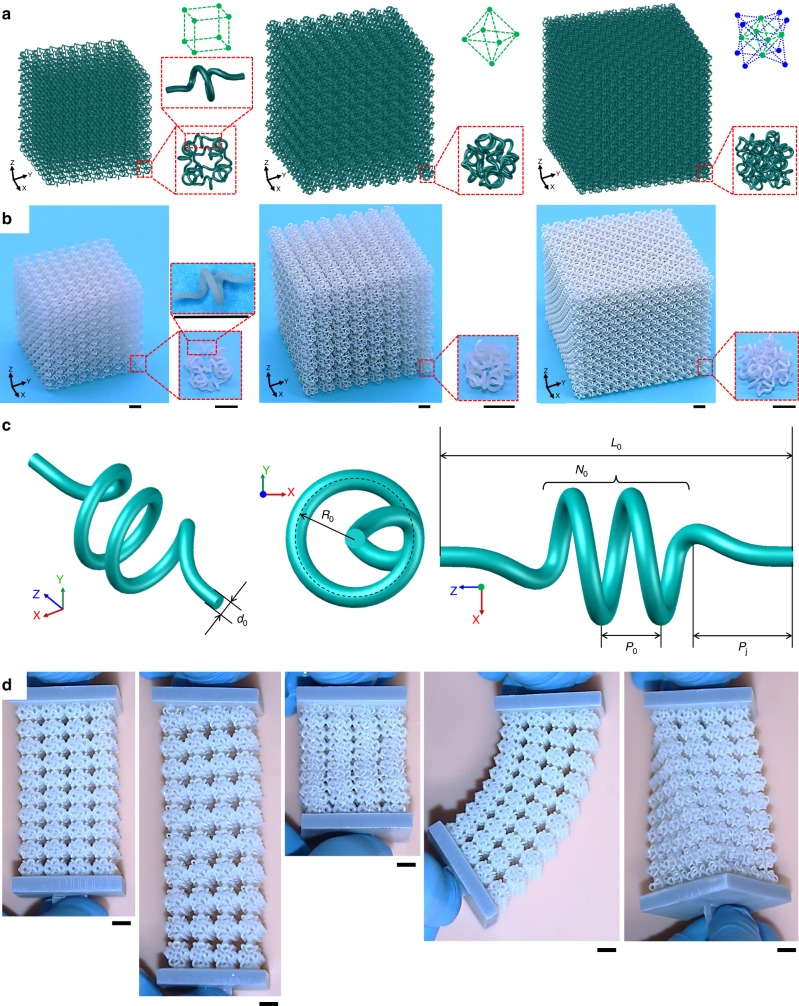
Design and manufacture of soft 3D network materials. **a** Conceptual illustration of the microstructure design for soft 3D network materials with three different lattice topologies (from left to right: cubic, octahedral, and octet). The filamentary microstructure with a 3D helical configuration serves as the building block that connects the lattice nodes. **b** Optical images of the specimens fabricated using the polyjet 3D printing technique, according to the designs in **a**. Each specimen consists of 8 × 8 × 8 unit cells. **c** Schematic illustration of the geometric parameters associated with the 3D helical microstructure. *d*_0_, *R*_0_, *N*_0_, *p*_0_, and *p*_j_ denote the diameter of the fiber, the radius of the helix, the number of the coil, the pitch, and the joint length, respectively. **d** Optical images of a representative soft 3D network structure before and after large levels of deformations (stretching, compression, bending, and twisting). This structure is composed of 4 × 4 × 10 unit cells, each with an octahedral lattice topology. Scale bars, 5 mm in **b** and **d**.

Figure 1 presents the conceptual designs and excellent deformability of the bio-mimetic, soft 3D network materials. Inspired by the network constructions and helical microstructures of many collagenous tissues, we develop a 3D network design by exploiting a type of 3D helical microstructures as the building blocks that are extended with different 3D lattice topologies. Figure 1a provides a schematic illustration of soft 3D network materials with three different lattice topologies (cubic, octahedral, and octet). In particular, the helix-shaped filamentary microstructure (Fig. 1c) consists of three segments, including a central part that corresponds to a circular helix and two connection parts that avoid tangling of the microstructures at the nodal regions of the network. The parametric equation of the central line associated with the helical microstructure is provided in Supplementary Note 1, which can ensure the continuity of the tangent line at the two ends. The geometry of the helical microstructure is fully characterized by four dimensionless parameters, including the normalized diameter (*d*_0_/*R*_0_) of the fiber, the number of the coil *N*_0_, the normalized pitch (*p*_0_/*R*_0_), and the normalized joint length (*p*_j_/*R*_0_), where *R*_0_ is the radius of the helix. The end-to-end distance of the helical microstructure can be given by *L*_0_ = 2*p*_j_ + *N*_0_*p*_0_. To avoid evident stress concentration at the nodal connections, a spherical lattice node with spatial rounding is adopted (see Supplementary Fig. 1 for details). It is noteworthy that the total numbers of connected filaments per node are 6, 8, and 12 for cubic, octahedral, and octet topologies, respectively, suggesting the octet lattice topology as the most densely distributed network.

Figure 1b presents optical images of three representative network materials fabricated using polyjet 3D printing techniques (Object EDEN260VS, Stratasys, MN, USA), according to the designs in Fig. 1a. Considering the resolution (layer thickness ~16 µm) of the exploited polyjet 3D printing instrument and the circular cross section, the fiber diameter *d*_0_ is set as ~400 µm in most of the experiments in this study. Other dimensionless geometric parameters of the helical microstructures in Fig. 1b are given by *p*_0_/*R*_0_ = 1, *d*_0_/*R*_0_ = 0.35, *N*_0_ = 1, and *p*_j_/*R*_0_ = 2. This type of soft 3D network materials offers excellent deformability under various forms of mechanical deformations. Taking the soft octahedral network material as an example, Fig. 1d shows the optical images before and after large levels of deformations, such as the stretching (applied strain *ε* ≈ 55%), compression (applied strain *ε* ≈ 33%), bending (curvature radius ≈ 15*L*_0_), and twisting (applied twisting angle ≈ 90°). The detailed deformation process is provided in Supplementary Movie 1.

**Fig. 2:**
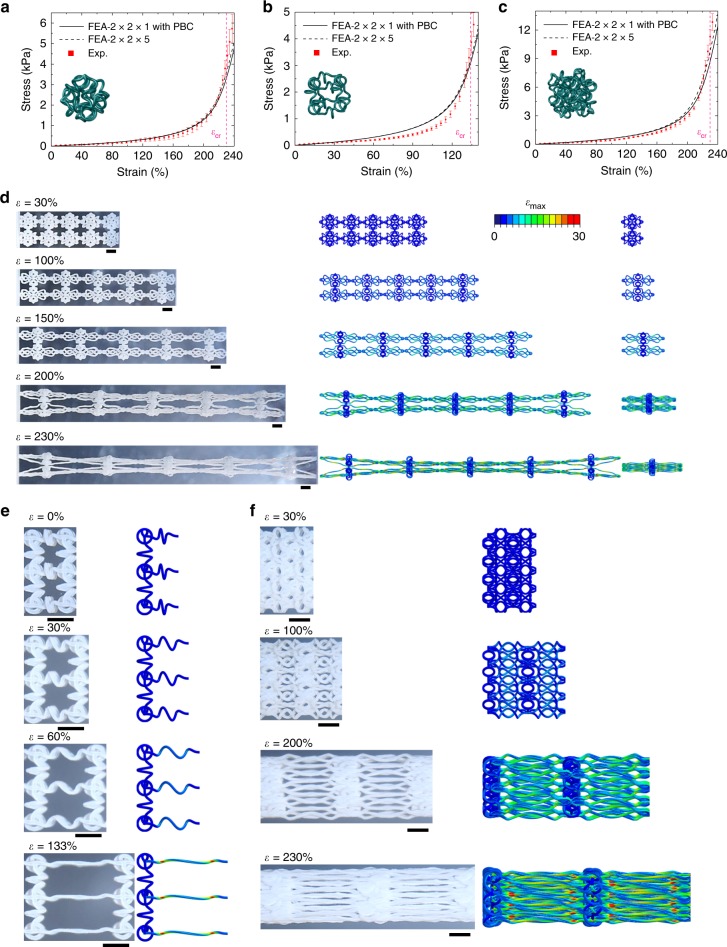
Nonlinear mechanical responses and microstructure deformations. **a**–**c** Experimental and FEA results of stress–strain curves of three soft 3D network materials with the same helical microstructures and different lattice topologies (cubic, octahedral, and octet). The experimental results based on samples with 2 × 2 × 5 unit cells are denoted by red rots, and the FEA results based on unit segments with 2 × 2 × 1 unit cells, and periodic boundary conditions are denoted by black solid lines. For the octahedral lattice, the FEA results of the entire sample with 2 × 2 × 5 unit cells are included for comparison, as denoted by the black dashed line. **d** Optical images (left) of a soft octahedral network structure under different levels of stretching, and the corresponding FEA results (middle and right) based on the entire sample and the unit segment (2 × 2 × 1 unit cells) with periodic boundary conditions. **e** Experimental and FEA images of the middle segment in a soft cubic network structure under different levels of stretching. **f** Similar results for a soft octet network structure. All error bars in this figure and the followings are standard deviations. Scale bars, 5 mm in **d**–**f**.

Figure 2 presents the results of combined experimental measurements and finite element analyses (FEA; see Methods section for details) on the nonlinear mechanical responses of soft 3D network materials under uniaxial stretching. Figure 2a, d shows the J-shaped stress–strain curve of the soft octahedral network material and associated microstructure deformations. At the first stage of stretching (*ε* < 200%), the helical microstructures mainly undergo combined spatial bending and twisting deformations, as well as rotational motions to align with the loading direction. The stress–strain curve increases relatively slowly during this stage. With the further increase of the applied strain, the tension of helical microstructures come into play and gradually dominates the deformations, leading to a sharp increase of the stress and the formation of a J-shaped stress–strain curve similar to that of biological tissues. The critical strain (*ε*_cr_) that marks the transition of deformation mode in the network material can be estimated by the condition when the helical microstructure is fully extended and aligned with the loading direction. In particular, the critical strain (*ε*_cr_) of soft octahedral network material is given by $\varepsilon _{{\mathrm{cr}}\left( {{\mathrm{Octahedral}}} \right)} = \sqrt 2 L_{{\mathrm{arc - length}}}/L_0 - 1$, where *L*_arc−length_ is the total arc length of a helical microstructure and can be derived as

1 $$L_{{\mathrm{arc}}\,-\,{\mathrm{length}}} = \left( {N_0\sqrt {1 + 4\pi ^2R_0^2/p_0^2} + 2l_{\mathrm{j}}/p_0} \right)L_0/\left( {N_0 + 2p_{\mathrm{j}}/p_0} \right)$$

where *l*_j_ is the total arc length of each joint at the end of the helical microstructure (see Supplementary Note 1 for details). For the network geometry shown in Fig. 2d, the critical strain is determined as ~230%, as marked by the pink dashed line in Fig. 2a. The microstructures are fully straightened at *ε* = *ε*_cr_ (Fig. 2d), resulting in a substantially increased tangential modulus (*E*_cr_ = 12.65 kPa) as compared to the elastic modulus (*E*_elastic_ = 0.19 kPa) at the initial state. Both of the FEA based on the full-scale network sample (with 2 × 2 × 5 unit cells) and the unit segment (with 2 × 2 × 1 unit cells and periodic boundary conditions) are performed. Here, the periodic boundary conditions require the two boundary surfaces to be in the same shapes during deformations, and do not fix those surfaces as planes. The calculated stress–strain curves show good agreements with each other, as well as with the experimental results (Fig. 2a). The deformed configurations predicted by the unit segment model are close to that of the central segment in the experiment and full-scale FEA, although slight differences of the lateral deformations can be observed, due to the finite boundary effect. These results indicate that the FEA based on the unit segment model offer accurate predictions of the mechanical responses.

Figure 2b, e and Supplementary Fig. 2a show the J-shaped stress–strain curve of the soft cubic network material and associated microstructure deformations. The helical microstructures are exactly the same as those in the soft octahedral network material discussed above. In this case, a set of helical microstructures are aligned with the loading direction at the initial state, such that those microstructures undergo only bending and twisting deformations to achieve the full unraveling, without the need to experience rotational motions. The resulting critical strain is identical to that of helical microstructures, as given by $\varepsilon _{{\mathrm{cr}}\left( {{\mathrm{Cubic}}} \right)} = \varepsilon _{{\mathrm{cr}}\left( {{\mathrm{Helix}}} \right)} = L_{{\mathrm{arc - length}}}/L_0 - 1$, with the arc length shown in Eq. (1). There is almost no lateral shrinkage as the applied strain reaches the critical strain (~133%), due to the essentially decoupled deformations of the helical microstructures along different directions. Note that the uniaxial stretching of a single helical microstructure yields a quite similar J-shaped stress–strain curve (see Supplementary Figs. 3 and 4 for details). Figure 2c, f and Supplementary Fig. 2b show the results for the soft octet network material with the same helical microstructures in the octahedral and cubic network materials. Because of the similar alignment of helical microstructures, the critical strain is the same as that of the octahedral network, i.e., $\varepsilon _{{\mathrm{cr}}\left( {{\mathrm{Octet}}} \right)} = \varepsilon _{{\mathrm{cr}}\left( {{\mathrm{Octahedral}}} \right)} = \sqrt 2 L_{{\mathrm{arc - length}}}/L_0 - 1$. In comparison to the octahedral network, the addition of tetragonal components and associated microstructures in the octet network increases the stiffness substantially, as evidenced by the critical stress (~10.32 kPa vs. 3.45 kPa) at *ε*_cr_. Additionally, the cross-sectional shrinkage is also substantially reduced, as shown by the deformed configurations at *ε* = 230% (Fig. 2c, f). In all of those cases, the FEA results almost agree very well with the experimental results, indicating the FEA as a reliable tool of design optimization to achieve desired mechanical responses.

**Fig. 3:**
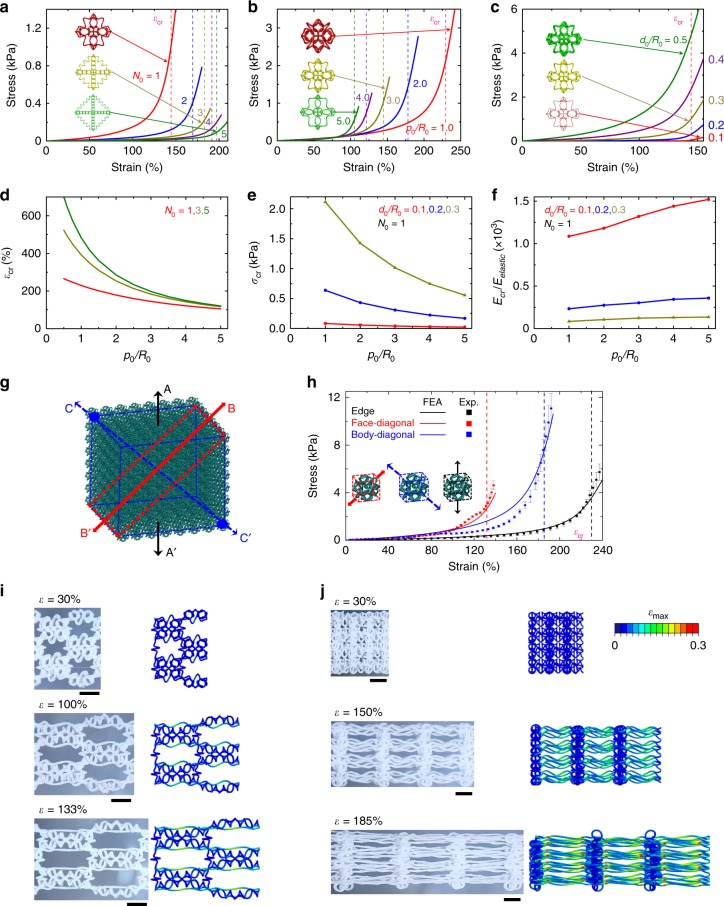
Design rules and mechanical responses along different characteristic directions. **a**–**c** Effects of three key dimensionless geometric parameters (the number *N*_0_ of the coil, the normalized pitch *p*_0_/*R*_0_, and the normalized fiber diameter *d*_0_/*R*_0_) on the J-shaped stress–strain curves of soft octahedral network materials. **d** Critical strain of the J-shaped stress–strain curve vs. the normalized pitch *p*_0_/*R*_0_ for different numbers *N*_0_ of the coil. **e**, **f** Effective critical stress and modulus ratio *E*_cr_/*E*_elastic_ vs. the normalized pitch *p*_0_/*R*_0_ for different normalized fiber diameters *d*_0_/*R*_0_. **g** Schematic diagram of soft octahedral network materials under uniaxial tension along the edge, face-diagonal, and body-diagonal directions, denoted by A–A′, B–B′, and C–C′, respectively. **h** Experimental and FEA results of J-shaped stress–strain curves for soft octahedral network materials under uniaxial tension along the edge, face-diagonal, and body-diagonal directions. **i** Experimental and FEA images of the middle segment in a soft octahedral network structure under different levels of uniaxial stretching along the face-diagonal direction. **j** Similar results under uniaxial tension along the body-diagonal direction. Scale bars, 5 mm in **i** and **j**.

Figure 3a–f elucidates the microstructure–property relationship and illustrates the influences of key geometric parameters on the J-shaped stress–strain curves. Here, we focus on soft octahedral network materials constructed with a range of different helical geometries, as characterized by three dimensionless parameters (*N*_0_, *d*_0_/*R*_0_, and *p*_0_/*R*_0_), noting that the normalized joint length *p*_j_/*R*_0_ plays a relative minor effect on the mechanical response. Figure 3a presents J-shaped stress–strain curves calculated by FEA for five different coil numbers (*N*_0_), by fixing the normalized pitch (*p*_0_/*R*_0_ = 3) and the normalized fiber diameter (*d*_0_/*R*_0_ = 0.3), where the critical strain (*ε*_cr(Octahedral)_) is marked by dashed lines. The stress–strain curve shifts downward and rightward gradually with increasing the coil number (*N*_0_), because the helical microstructures become more slender. The critical strain gradually approaches a stable value with the further increase of *N*_0_, in accordance with Eq. (1). Figure 3b, c shows the effects of the normalized pitch (*p*_0_/*R*_0_) (by fixing *N*_0_ = 1 and *d*_0_/*R*_0_ = 0.3) and the normalized fiber diameter (*d*_0_/*R*_0_) (by fixing *N*_0_ = 1 and *p*_0_/*R*_0_ = 3), respectively. Both of the critical strain and the critical stress decrease with the increase of *p*_0_/*R*_0_. Differently, the normalized fiber diameter (*d*_0_/*R*_0_) mainly affects the critical stress and the sharpness of transition across the critical strain, and the variation can be observed more clearly in Supplementary Fig. 5.

The J-shaped stress–strain curve is mainly characterized by three key quantities, including the critical strain (*ε*_cr_) that marks the transition point in the stress–strain response, the critical stress (*σ*_cr_) at *ε*_cr_ that is of relevance to the strength of the network material, and the ratio (*E*_cr_ / *E*_elastic_) of tangential modulus at *ε*_cr_ to the initial elastic modulus that measures the sharpness of transition. According to Eq. (1), the critical strain (*ε*_cr_) depends highly on the normalized pitch (*p*_0_/*R*_0_) and the number (*N*_0_) of coil, when the joint length (*p*_j_/*R*_0_) is fixed. Figure 3d shows such a dependence, where *ε*_cr_ increases at a decreased *p*_0_/*R*_0_ or an increased *N*_0_. Generally, the elastic moduli of the stretching-dominated and bending-dominated 3D lattice materials scale with the relative density ($\bar \rho$) and its square ($\bar \rho ^2$), respectively^64^. Therefore, the modulus ratio (*E*_cr_/*E*_elastic_) follows approximately the scaling of $E_{{\mathrm{cr}}}/E_{{\mathrm{elastic}}} \propto \left( {d_0/R_0} \right)^{ - 2}$, noting that $\bar \rho \propto \left( {d_0/R_0} \right)^2$, $E_{{\mathrm{cr}}} \propto \left( {d_0/R_0} \right)^2$, and $E_{{\mathrm{elastic}}} \propto \left( {d_0/R_0} \right)^4$, for small relative densities (e.g., $\bar \rho < 20\%$). This scaling suggests a sharper transition in the J-shaped stress–strain curve for network materials with a smaller normalized diameter (*d*_0_/*R*_0_). In the representative case of a single coil in the helical microstructure (i.e., *N*_0_ = 1), the critical stress (*σ*_cr_) and the modulus ratio (*E*_cr_/*E*_elastic_) are plotted against the normalized pitch (*p*_0_/*R*_0_) for a range of fiber diameters (*d*_0_/*R*_0_), as presented in Fig. 3e, f. The increase of *p*_0_/*R*_0_ or decrease of *d*_0_/*R*_0_ leads to a more slender and flexible helical microstructure, thereby resulting in a reduction of the critical stress (*σ*_cr_) and an enhancement of the modulus ratio (*E*_cr_/*E*_elastic_). Additional results of J-shaped stress–strain curves with different geometric parameters appear in Supplementary Figs. 6 and 7.

### Effects of loading angles and randomly distributed defects

Many 3D biological tissues display anisotropic mechanical responses under large levels of stretching^65–67^. Figure 3g–j presents the anisotropic mechanical behavior in the soft 3D network materials developed in this work. Taking the octahedral network material as an example, we investigate the nonlinear mechanical responses under uniaxial stretching along the face-diagonal (B–B′) and body-diagonal (C–C′) directions (Fig. 3g and Supplementary Fig. 8), in addition to the principal direction (i.e., edge direction (A–A′)) studied in Fig. 2a, d. These three directions represent the most characteristic loading directions for the octahedral lattice topology with cubic symmetry. Figure 3h shows the measured and predicted stress–strain curves along these directions, where the initial alignment of the microstructures plays a critical role. For the uniaxial stretching along the face-diagonal (B–B′) direction, a set of helical microstructures are aligned with the loading direction at the initial state, such that these microstructures accommodate a majority of the applied load, as evidenced by the microstructure deformations (Fig. 3i). In this case, the critical strain (~133%) is equal to that of helical microstructures. For the uniaxial stretching along the body-diagonal (C–C′) direction, a half portion of helical microstructures shows the same angle (~35.3^°^) with respect to the loading direction, while the other half stays in a plane perpendicular to the loading direction (Fig. 3j). In this case, the critical strain can be calculated by $\frac{{\sqrt 6 }}{2}\left( {\varepsilon _{{\mathrm{cr}}\left( {{\mathrm{Helix}}} \right)} + 1} \right) - 1$, and is determined as 185% for *ε*_cr(Helix)_ = 133%. In all of the three different loading conditions, the J-shaped stress–strain curves and deformed configurations predicted by FEA always agree reasonably well with experimental results.

**Fig. 4:**
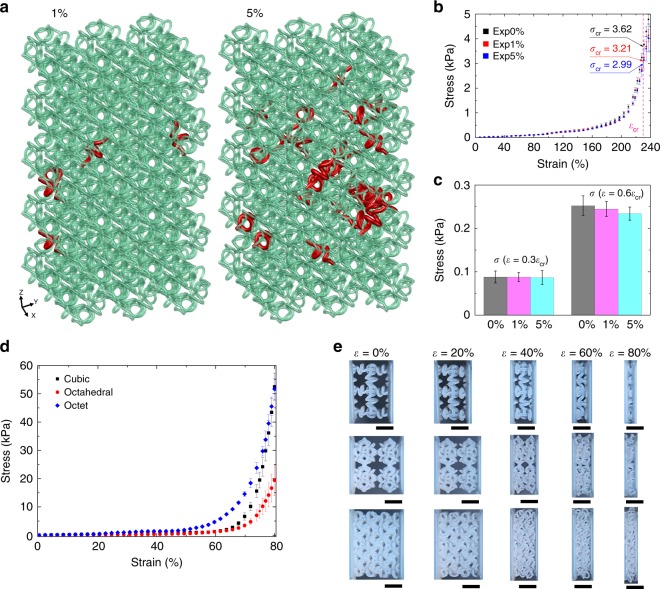
Stress–strain curves in the presence of random defects or under uniaxial compression. **a** Visualization the randomly distributed defects (i.e., missing microstructures) in soft octahedral network materials with two typical defect densities (1% and 5%). **b** J-shaped stress–strain curves of soft octahedral network materials with 0%, 1%, and 5% random defects. For each level of defect density, at least three samples with different distributions of random defects are taken into account. **c** Influence of the randomly distributed defects on the effective critical stress. **d** Measured stress–strain curves under uniaxial compression, for soft 3D network materials with three different topologies (cubic, octahedral, and octet). All of the structures consist of 2 × 2 × 2 unit cells and the same helical microstructures as those in Fig. 2. **e** Optical images of the three soft network structures under different levels of uniaxial compression. Scale bars, 5 mm in **e**.

Figure 4a–c illustrates the influence of defects on the J-shaped stress–strain response of soft 3D network materials. Here, we focus on an extreme type of defects in the form of missing microstructures that are randomly distributed in the network material with prescribed densities. Figure 4a and Supplementary Fig. 9 provide two representative samples of imperfect octahedral network materials with defect densities of 1% and 5%, where the randomly distributed defects are visualized (in red). Figure 4b shows the J-shaped stress–strain curves of octahedral network materials along the principal directions, in the case of 0%, 1%, and 5% random defects. For each prescribed defect density, three samples with different defect distributions are fabricated and tested. Slight reduction of the stress response at small and moderate levels of applied strains (e.g., <140%) is observed for defect density up to 5%, indicating that the octahedral network material is not very sensitive to the appearance of missing microstructures. As an evidence, the effective stresses at *ε* = 0.3*ε*_cr_ and 0.6*ε*_cr_ decrease by ~3% and 7%, respectively, for the defect density of 5%, as shown in Fig. 4c. In the case of 1% defect density, the relative reductions (~1% and 3%) of the effective stresses at *ε* = 0.3*ε*_cr_ and 0.6*ε*_cr_ are even smaller.

Figure 4d, e illustrates the compressive mechanical responses of soft 3D network materials with cubic, octahedral, and octet topologies. Here, we exploited the samples consisting of 2 × 2 × 2 unit cells, instead of those with 2 × 2 × 5 unit cells that tend to occur global buckling during uniaxial compression (Supplementary Fig. 10). At the initial stage of uniaxial compression, the stress increases slowly with the increase of strain, as the helical microstructures are governed by bending and twisting deformations. With the further increase of compressive strain, the helical microstructures begin to contact with the neighboring microstructures, leading to the stiffening of the material. Such a densification process can proceed until almost all of helical microstructures are flattened (*ε* ≈ 80%). As a result, the soft 3D network materials also show the J-shaped stress–strain response under uniaxial compression, although the deformation mechanism is different from of uniaxial stretching. It is noteworthy that the octet network material also offers the highest stiffness under compression, due to the most densely arranged microstructures.

### Demonstrative applications of soft 3D network materials

**Fig. 5:**
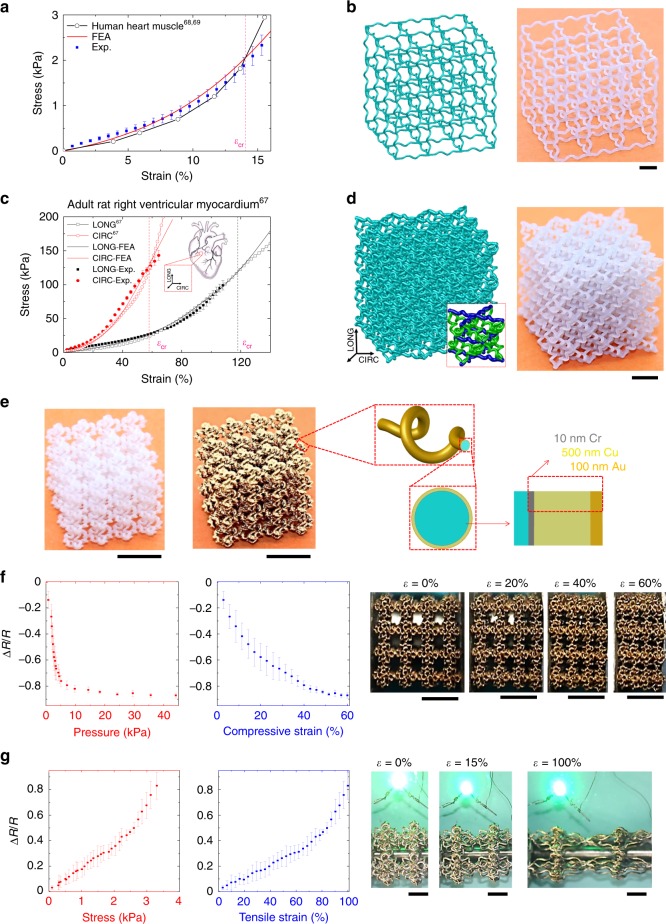
Bio-mimetic designs and demonstrative applications. **a** Typical passive stress–strain curves of the human heart muscle^68,69^, artificial network materials, with corresponding FEA results for the latter. Here, the mechanical testing of artificial network materials was performed at 25 °C. **b** Geometric model and optical image of the artificial network material in **a**. **c** Stress–strain curves of right ventricular myocardium of adult rat^67^ along the circumferential and longitudinal directions, along with the results of artificial network materials and corresponding FEA results. Here, the mechanical testing of artificial network materials was performed at 23 °C. **d** Geometric model and optical image of the artificial network material in **c**. **e** Optical images of a soft octahedral network before and after magnetron sputtering. A thin coating of 10 nm Cr, 500 nm Cu, and 100 nm Au (from inside to outside) is generated by the sputtering. **f** Relative resistance change and effective pressure measured during the uniaxial compression of soft octahedral network materials, along with the deformed configurations. **g** Relative resistance change and effective tensile stress measured during the uniaxial stretching of soft octahedral network materials, along with the deformed configurations. A green LED connected with the stretched conducting network serves as a demonstration. Scale bars, 5 mm in all images.

The rational 3D network design introduced above allows the development of artificial materials that can reproduce nonlinear stress–strain curves of 3D biologic tissues. For example, Fig. 5a, b presents a cubic network material whose J-shaped stress–strain curve is very close to that of the human heart muscle^68,69^. Note that the source data in Nagueh et al ^68^ is given as stress vs. sarcomere length (*L*_S_), where the sarcomere length can be converted into the engineering strain by *ε* = (*L*_S_ − *L*_initial_)/*L*_initial_, with *L*_initial_ denoting the initial length. Considering the relative low transition strain (i.e., critical strain *ε*_cr_ ≈ 14%) of the stress–strain curve, we exploit the cubic lattice to construct the artificial network material. Utilizing the FEA as a tool to predict the stress–strain curves, we follow a design procedure outlined in Supplementary Fig. 11 and Supplementary Note 2 to determine the geometric parameters (*p*_0_/*R*_0_ = 8.5, *d*_0_/*R*_0_ = 1.05, *N*_0_ = 1, and *p*_j_/*R*_0_ = 5) of the 3D network material. The artificial material with these microstructure parameters is fabricated (Fig. 5b) and tested under uniaxial stretching. The J-shaped stress–strain curve measured based on the artificial material matches well with the target one of the human heart muscle, as well as the FEA predictions.

Some biological tissues exhibit anisotropic mechanical properties, posing more difficulties to the development of artificial materials with similar stress–strain responses. Figure 5c shows an octet network material, whose microstructure geometries can be tailored to reproduce the stress–strain curves of an adult rat right ventricular myocardium^67^, along both the circumferential and longitudinal directions. In this case, we exploited two different types of helical microstructures within the representative unit cell (marked as blue and green in the inset of Fig. 5d), such that the different critical strains (*ε*_cr(Circumferential)_ ≈ 58% and *ε*_cr(Longitudinal)_ ≈ 118%) along the circumferential and longitudinal directions can be reproduced simultaneously. Considering the partially decoupled mechanical responses along the two perpendicular directions, the design procedure consists of a two-stage process that firstly focuses on matching the target stress–strain curve along the circumferential direction by adjusting the geometric parameters of an octet network with a single type of helical microstructures. The second stage involves the replacement of certain helical microstructures (marked in blue in the unit cell, as in Fig. 5d) with other optimized geometries, in order to match the target stress–strain curve along the longitudinal direction. The optimal parameters are given by (*p*_0_/*R*_0_ = 3, *d*_0_/*R*_0_ = 0.9, *N*_0_ = 1, and *p*_j_/*R*_0_ = 3) for the green helical microstructures, and (*p*_0_/*R*_0_ = 8, *d*_0_/*R*_0_ = 2.9, *N*_0_ = 1, and *p*_j_/*R*_0_ = 7) for the blue helical microstructures in the inset of Fig. 5d. Figure 5c shows that the anisotropic stress–strain curves measured based on the artificial sample indeed match well with those of real rat right ventricular myocardium, for strains to ~65% (circumferential direction) and ~108% (longitudinal direction). To match the stress–strain curves over the entire strain ranges (~100 and 170% for the two directions, as in Engelmayr et al. ^67^), the base material of the 3D network should have a much higher elastic modulus and a sufficiently large strength, but this is unachievable based on the 3D printer (Object EDEN260VS, Stratasys, MN, USA) used in the current study.

The soft 3D network materials developed in this work hold promise for applications in bio-integrated devices. Figure 5e–g provides a demonstration of the conducting soft 3D network materials for potential uses as flexible pressure sensors and conductors. The magnetron sputtering allows the coating of metallic layers (10 nm Cr, 500 nm Cu, and 100 nm Au, from inside to outside) on all of the helical microstructures in the 3D network. Figure 5e presents optical images of an octahedral network sample before and after the magnetron sputtering, where the geometric parameters include *L*_0_ = 2 mm, *d*_0_ = 200 µm, *p*_0_/*R*_0_ = 3, *d*_0_/*R*_0_ = 0.7, *N*_0_ = 1, and *p*_j_/*R*_0_ = 2. To fabricate even smaller 3D network materials, other advanced manufacture techniques (e.g., two-photon lithography^9,70^) should be exploited. Under uniaxial compression, the relative resistance change and average pressure (i.e., uniaxial stress applied to the sample) are measured at different compressive strains, along with the deformed configurations, as shown in Fig. 5f. For the average pressure <5 kPa, the electrical resistance shows a high sensitivity to the pressure change, mainly due to the densification process that leads to a rapid increase of contact areas between the helical microstructures. When the average pressure is >20 kPa, most of the helical microstructures are in contact with each other, and thereby the resistance becomes insensitive to the pressure change. It is also interesting to note that the relative resistance change shows an approximately linear dependence on the compressive strain. These results suggest that the presented bio-mimetic network material is suitable for the detection of a small pressure (<5 kPa). Figure 5g (left and middle) presents the relative resistance change as a function of the tensile stress and tensile strain respectively, during the unaxial stretching of a relatively large conducting network (with *L*_0_ = 7.5 mm, *d*_0_ = 536 µm, *p*_0_/*R*_0_ = 3, *d*_0_/*R*_0_ = 0.5, *N*_0_ = 1, and *p*_j_/*R*_0_ = 2). The experimental results show a slight resistance change (<20% relatively) for the tensile strain up to ~40%, which can meet the requirements of certain practical applications. When connected with a commerical green light-emitting diode (LED), the light keeps turning on for tensile strain up to ~100% (the right panel of Fig. 5g and Supplementary Figs. 12–14).

## Discussion

In summary, this work reports a class of rational bio-mimetic 3D network designs for soft architected materials consisting of periodically arranged helical microstructures, with abilities to reproduce accurately the anisotropic, nonlinear stress–strain responses of 3D biological tissues. The developed soft 3D network materials exhibit a defect-insensitive mechanical behavior, and can offer tunable J-shaped stress–strain curves under both the tensile and compressive loadings. A comprehensive study based on experimental measurements and validated FEA elucidates the influences of geometrical parameters of helical microstructures and the lattice topology on the J-shaped stress–strain curves. When integrated with metallic layers through magnetic sputtering or atomic-layer deposition, the conducting 3D network materials can be potentially used as flexible pressure sensors and stretchable conductors, while offering bio-mimetic mechanical properties. When the base material of the 3D network is replaced by an even softer material (e.g., a mixture of elastomer and polymer), the resulting 3D network material can also offer a J-shaped stress–strain response (Supplementary Fig. 15). Collectively, these findings provide systematic guidelines for the 3D network design of architected materials and functional systems, with many application opportunities in bio-integrated electronic devices.

## Methods

### Fabrication of soft 3D network materials

Soft 3D network materials are fabricated using the polyjet 3D printing technique (Object EDEN260VS, Stratasys, MN, USA), which offers a minimum layer thickness of 16 μm during the 3D printing. The fabrication exploits two commerically available materials (VeroBlue and SUP707), in which the SUP707 is a water-soluble supporting material, while the VeroBlue is a digital polymeric material (as shown in Supplementary Fig. 16). The polyjet 3D printing allows the formation of predefined soft 3D network materials that are embedded in the support materials after the priniting. Immersing the printed hybrid structure into water dissolves the supporting materials, and completes the fabrication of network materials with desired 3D geometric configurations.

### Measurements of the stress–strain curve and resistance

The force–displacement curves were measured by a commercial mechanical testing machine, from which the stress–strain curves were obtained. A very low loading rate (~0.3 mm per minute) was adopted to ensure that the deformations are nearly quasi-static and the viscoelastic effect can be neglected. All the experiments of uniaxial tension and compression were performed by immersing the samples in a water box (300 mm × 400 mm × 380 mm) at a fixed temperature of 25 °C (except for that in Fig. 5c), considering that the mechanical properties of VeroBlue material is sensitive to the temperature change in the range of 20−30 °C. The experiments in Fig. 5c were performed at ~23 °C, such that the elastic modulus can be increased by approximately three times as compared to that at ~25 °C, and that the critical stress of the target stress–strain curve can be achieved based on the proposed network design. The electrical resistance of conducting 3D network materials (Fig. 5e–g) were measured at ~50 ^o^C at the different levels of applied strains in a thermostat mechanical testing machine. All of the experimental results were obtained based on the averages of at least three different individual samples. The photographs and video of soft 3D network materials at different deformations were recorded employing a digital camera (760D, Canon, Japan).

### Finite element analyses

The finite element analyses (FEA) were performed employing commercial software ABAQUS (SIMULIA, Providence RI) to calculate the deformations and stress–strain curves. Ten-node quadratic tetrahedron hybrid brick were adopted, and the meshes were refined to ensure the computational accuracy. For the polymeric material (VeroBlue) produced by the 3D printing, a hyperelastic constitutive relation following an incompressible Mooney–Rivlin law was adopted in the FEA. The two material parameters (*C*_10_ = 0.578 MPa and *C*_01_ = 1.364 MPa, following the denotations of commercial software ABAQUS) are determined by fitting the uniaxial stress–strain curve measured in experiments (Supplementary Fig. 16). Both full-scale models and periodic unit segments were exploited in the FEA. In order to save the computational cost based on the periodic unit segments, the displacement components along the uniaxial stretching direction are prescribed, and the boundaries are allowed to deform freely along the transverse direction. As shown in Fig. 2d and Supplementary Fig. 2, the unit segments in the middle of the network samples are approximately under periodic boundary conditions along the axial direction, as evidenced by the agreement of deformation results between the full-scale FEA and the FEA based on periodic unit segments. The effective stress (i.e., engineering stress) of the lattice material is defined as the total reaction force at the boundary divided by the initial cross-sectional area.

## Supplementary information

Supplementary Information

Description of Additional Supplementary Files

Supplementary Movie 1

## Data Availability

The data that support the findings of this study are available within the article and its Supplementary Information, and from the corresponding authors upon reasonable request.
