# Supplementary material for: Soft three-dimensional network materials with rational bio-mimetic designs
Source: Nat Commun. 2020 Mar 4;11:1180. doi: 10.1038/s41467-020-14996-5 (PMC7055264; doi:10.1038/s41467-020-14996-5)
Supplement: Supplementary file 1 — Supplementary Information [file 41467_2020_14996_MOESM1_ESM.pdf]

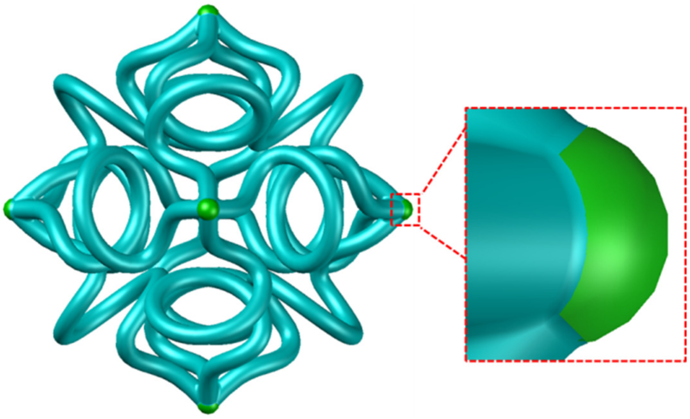

Supplementary Figure 1. Spherical lattice nodal connection with spatial rounding. Schematic illustration of nodal connection in the octahedral lattice topology. The diameter of the spherical node is adopted as 1.2 times the fiber diameter (d_0) in this study.

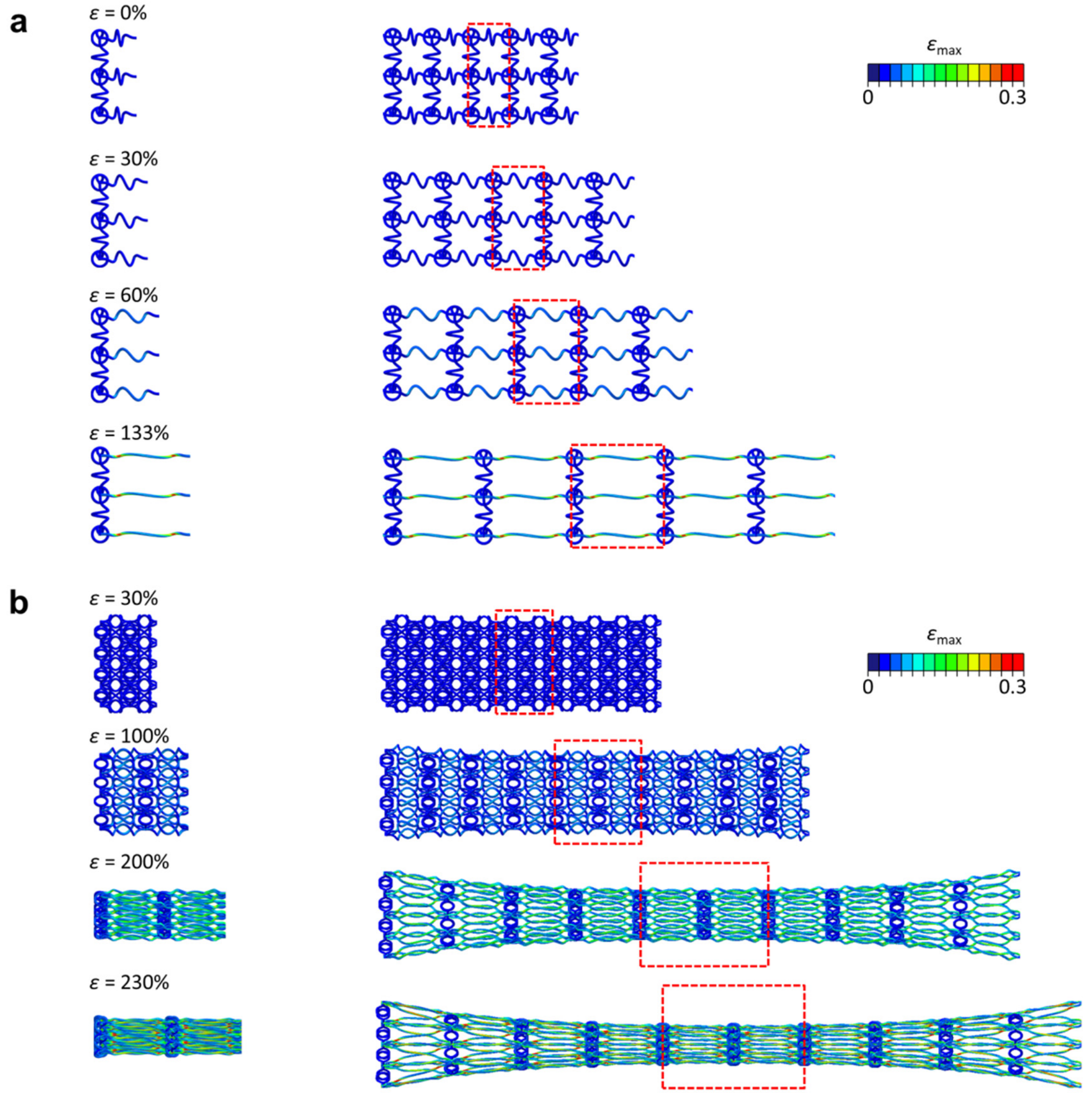

Supplementary Figure 2. Deformed configurations of soft cubic and octet network structures under stretching. (a) FEA results of a soft cubic network structure under different levels of stretching based on the unit segment (2 × 2 × 1 unit cells) with periodic boundary conditions (left) and the entire sample (right). (b) Similar results for a soft octet network structure.

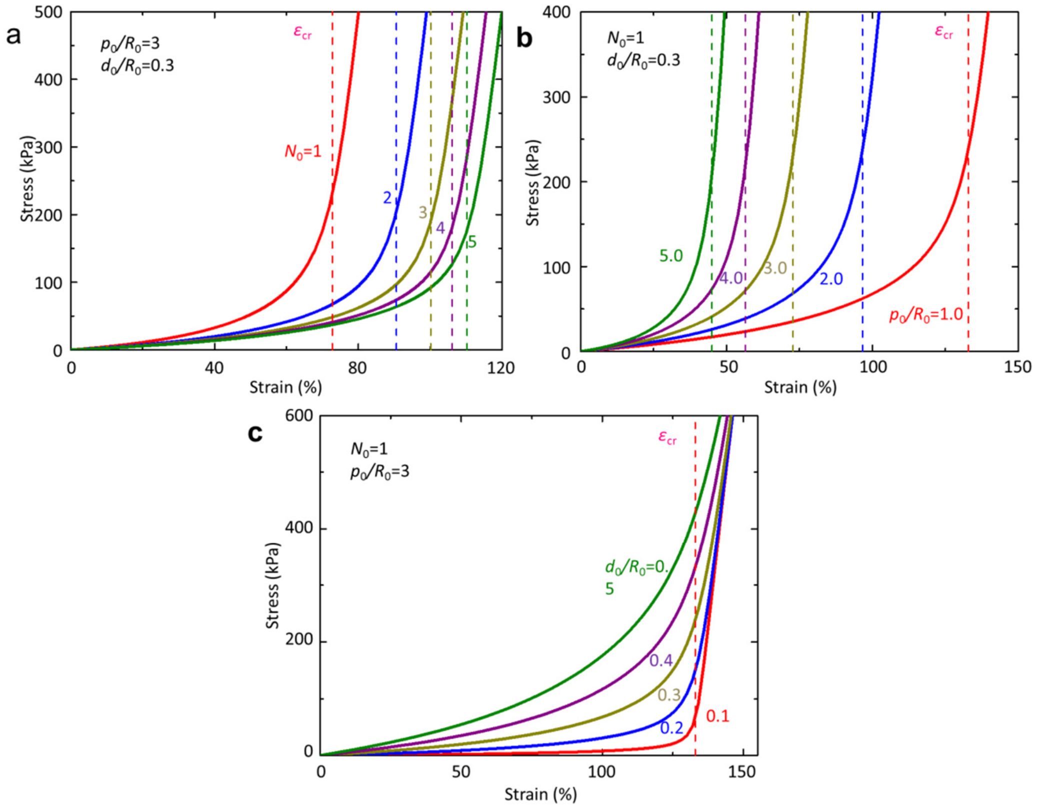

Supplementary Figure 3. Effects of three key dimensionless geometric parameters on J-shaped stress-strain curves of 3D helical microstructures. (a) The results for the helical geometries with five different coil numbers (N_0 =1, 2, 3, 4, 5). (b) The results for the helical geometries with five different normalized pitches (p_0 / R_0 = 1, 2, 3, 4, 5). (c) The results for the helical geometries with five different normalized fiber diameters (d_0 / R_0 = 0.1, 0.2, 0.3, 0.4, 0.5).

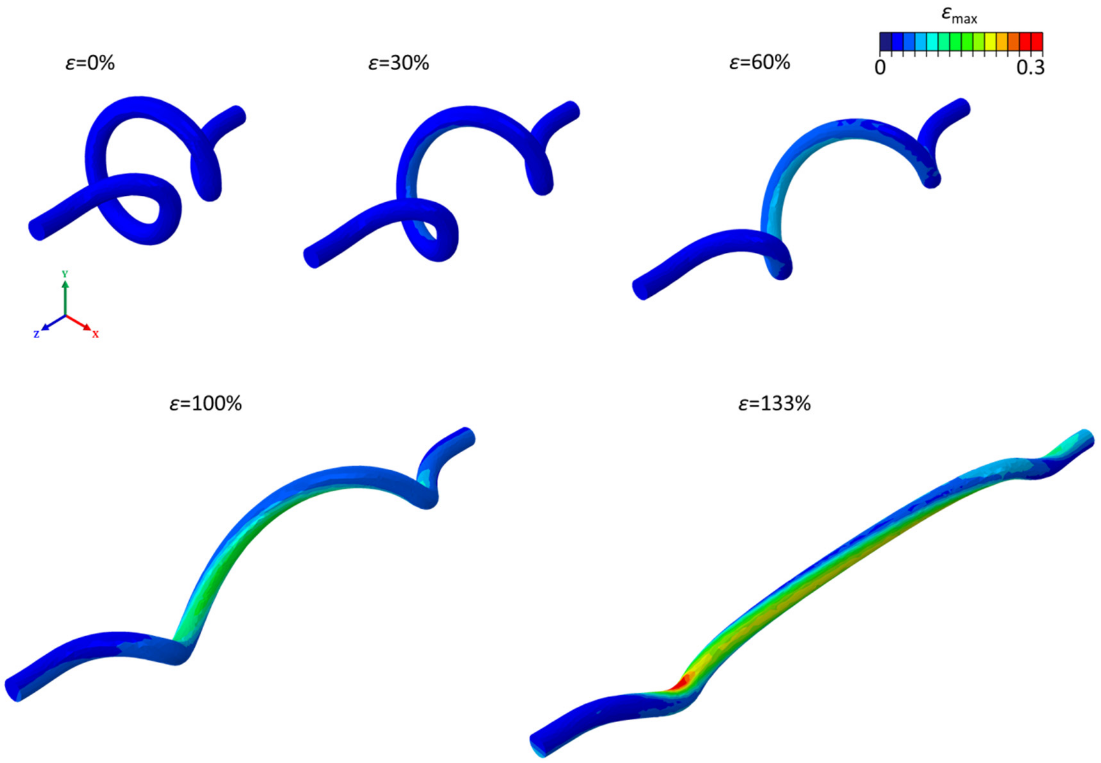

Supplementary Figure 4. Deformations of the helical microstructure under uniaxial stretching. Corresponding to soft 3D network materials in Fig. 2, the dimensionless geometric parameters are given by p_0 / R_0 = 1, d_0 / R_0 = 0.35, N_0 = 1 and p_j / R_0 = 2.

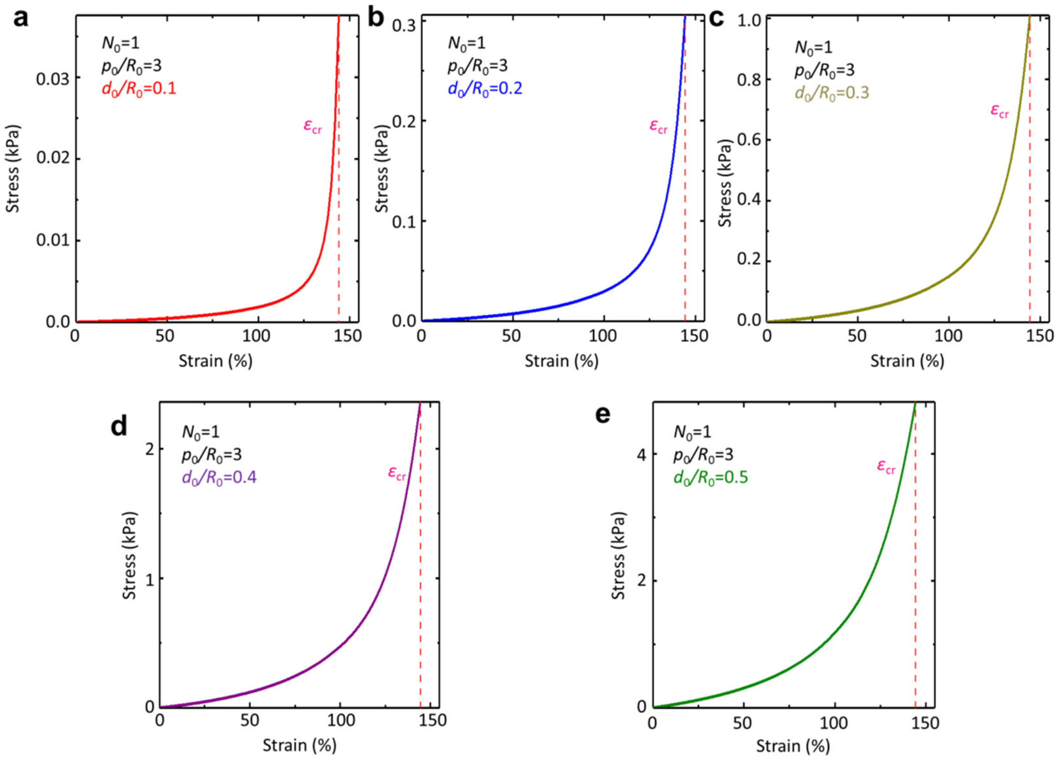

Supplementary Figure 5. Stress-strain curves of soft octahedral network materials in Fig. 3c. Stress-strain curves for five different normalized fiber diameters, (a) d_0 / R_0 = 0.1, (b) d_0 / R_0 = 0.2, (c) d_0 / R_0 = 0.3, (d) d_0 / R_0 = 0.4 and (e) d_0 / R_0 = 0.5, by fixing the normalized pitch (p_0 / R_0 = 3) and the coil number (N_0 =1).

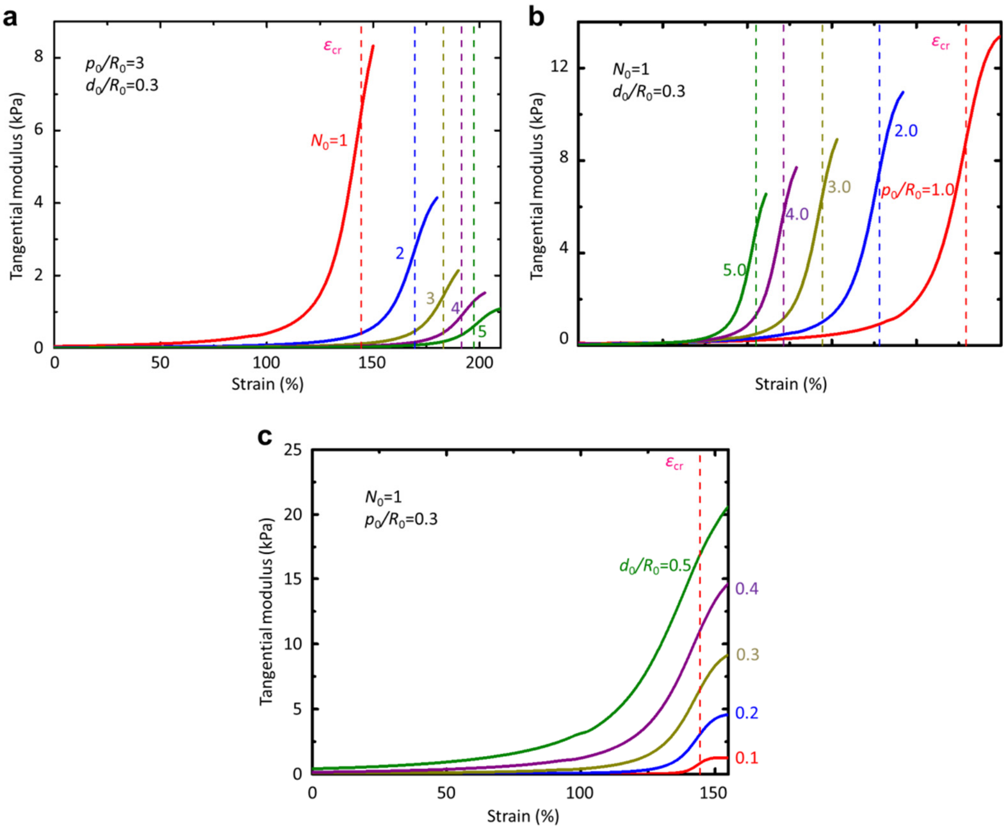

Supplementary Figure 6. Tangential modulus-strain curves of soft octahedral network materials in Fig. 3a.

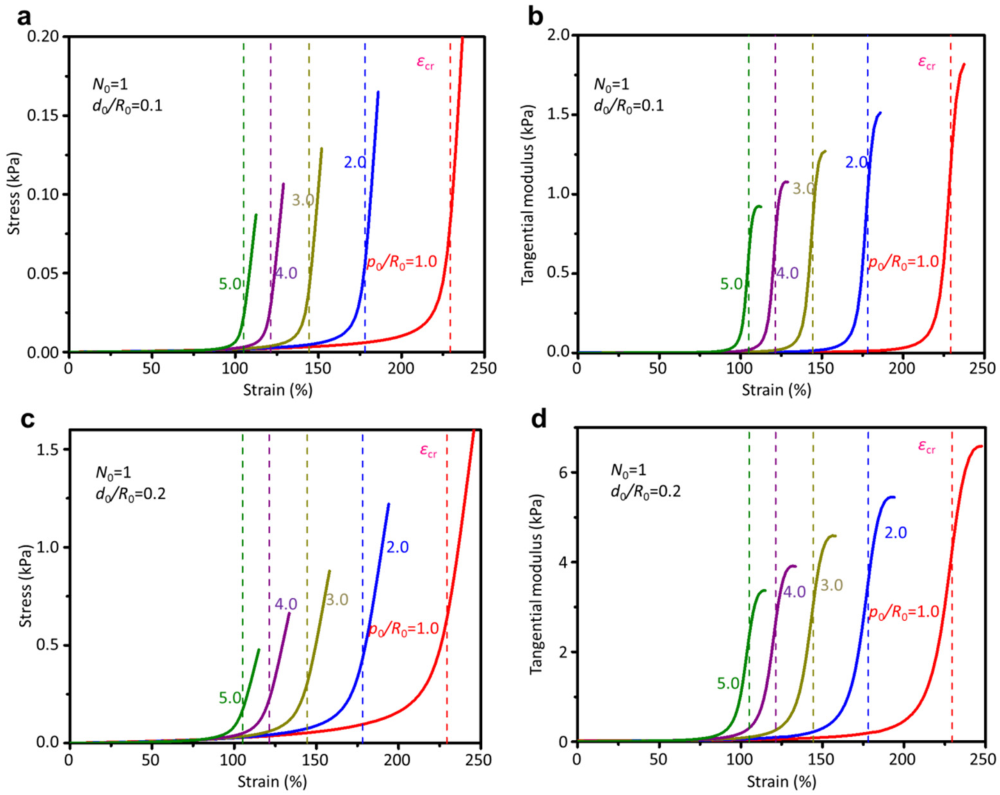

Supplementary Figure 7. Stress-strain curves and tangential modulus-strain curves of soft octahedral network materials in Fig. 3b. The critical stress (σ_{cr}) and ratio (E_{cr} / $E_{elastic}$) of tangential modulus at ε_{cr} to the initial elastic modulus are mentioned in Fig. 3b.

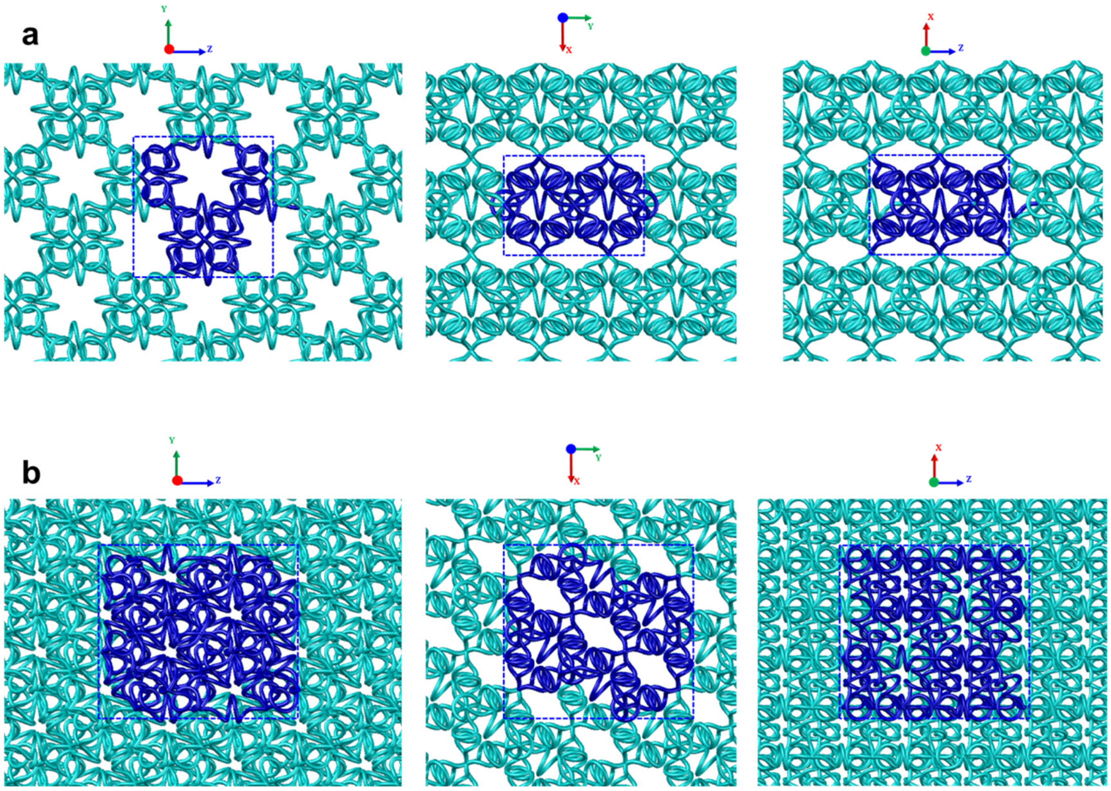

Supplementary Figure 8. Three different view angles of the face-diagonal and body-diagonal unit cell of the soft octahedral network materials. (a): face-diagonal, (b): body-diagonal. The stretching direction is assumed along the *y*-axis.

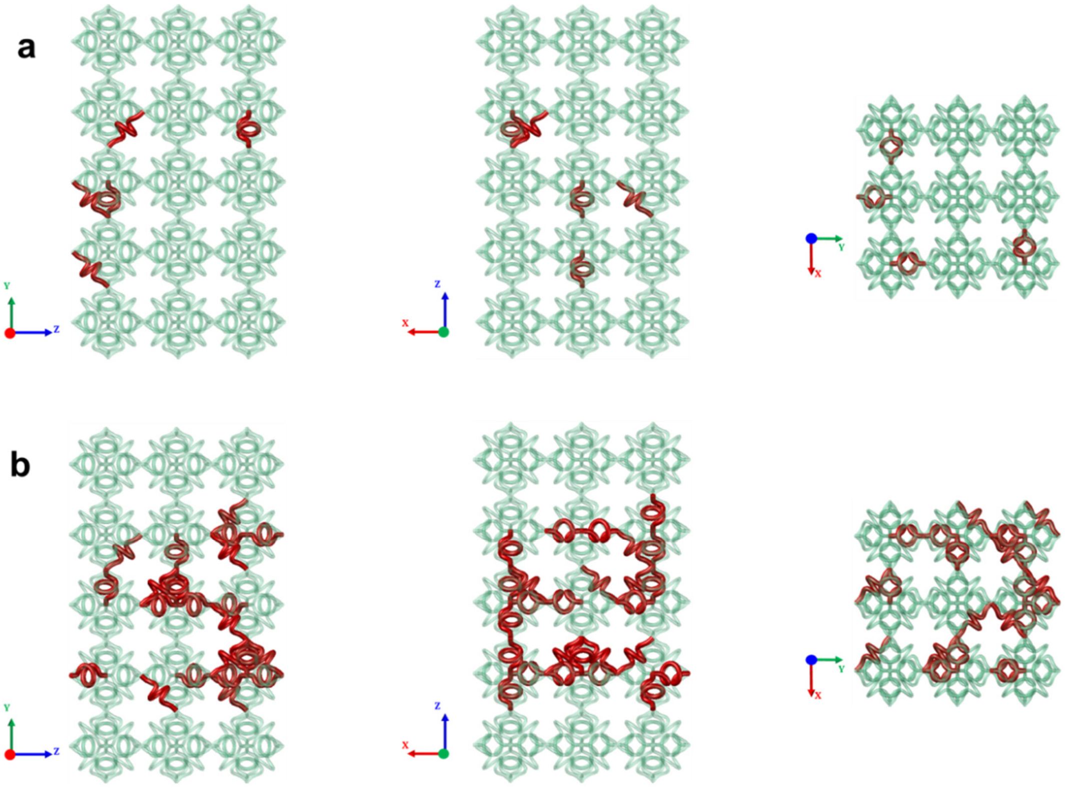

Supplementary Figure 9. Three different view angles of the 3D network samples with 1% and 5% random defects.

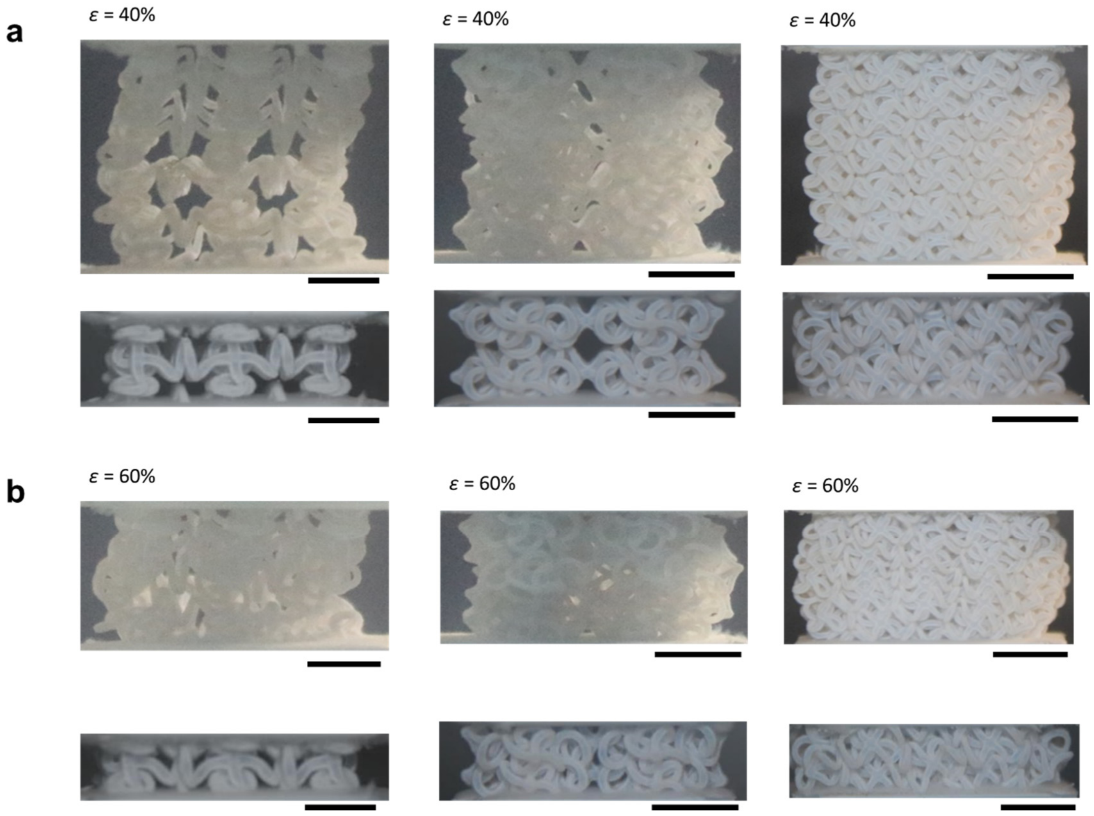

Supplementary Figure 10. Deformations of soft 3D network materials with 2 × 2 × 5 and 2 × 2 × 2 unit cells under uniaxial compression. (a) Deformations of soft 3D network materials with the cubic, octahedron and octet topologies, under the uniaxial compression of ε = 40%. (b) Similar results under the uniaxial compression of at ε = 60%.

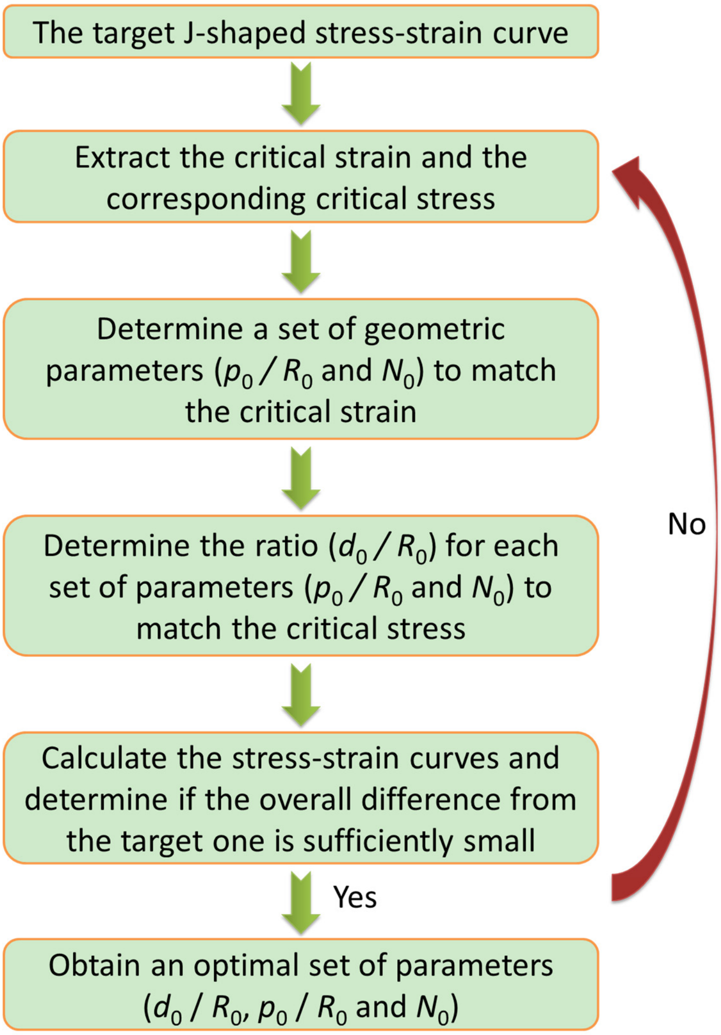

Supplementary Figure 11. Design procedure of soft 3D network materials to achieve desired stress-strain curves of biological tissues.

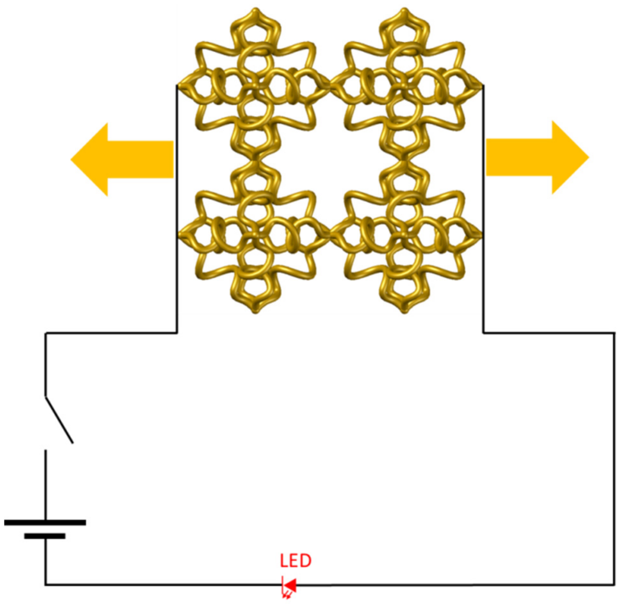

Supplementary Figure 12. Schematic diagram of the circuit design of the stretched conducting octahedral network materials connected with a LED.

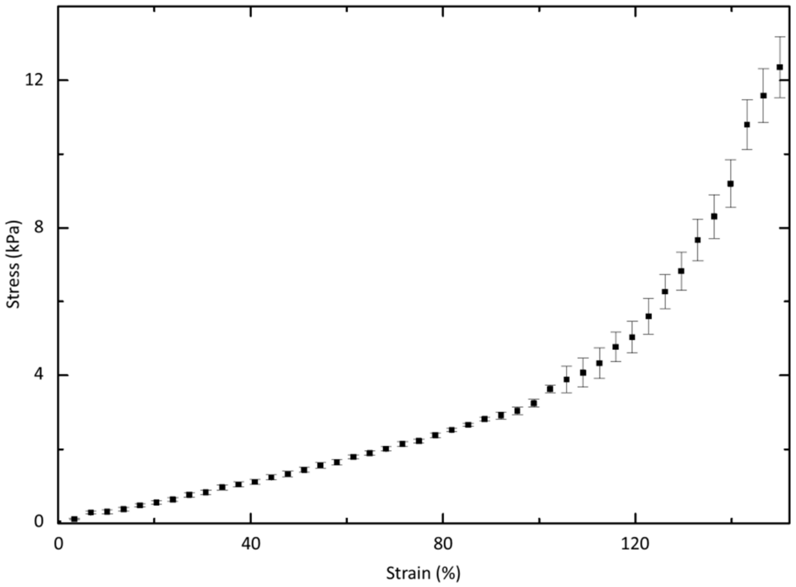

Supplementary Figure 13. **Stress-strain curves of soft octahedral network materials coated with a thin film metal by magnetron sputtering technique.**

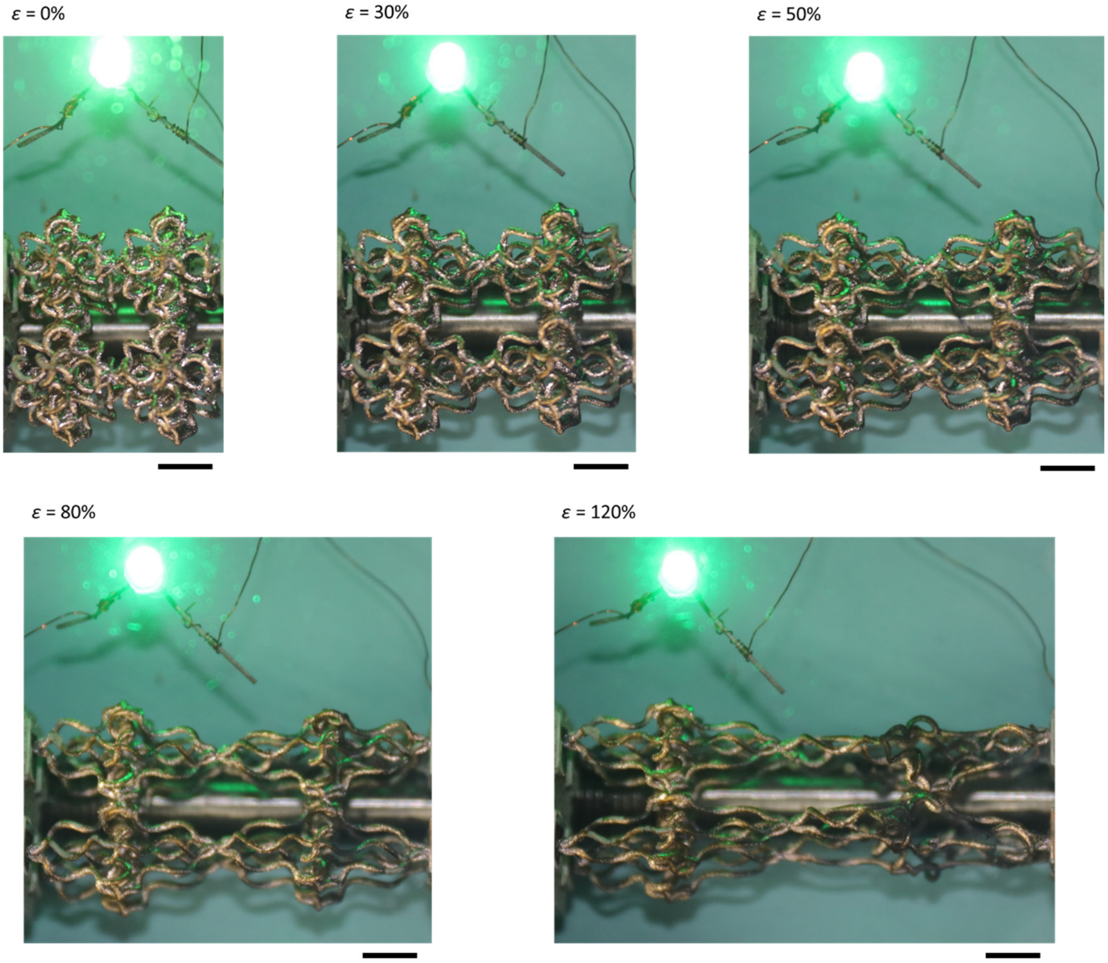

Supplementary Figure 14. Optical images of the stretching deformations in the conducting octahedral network material connected with a LED.

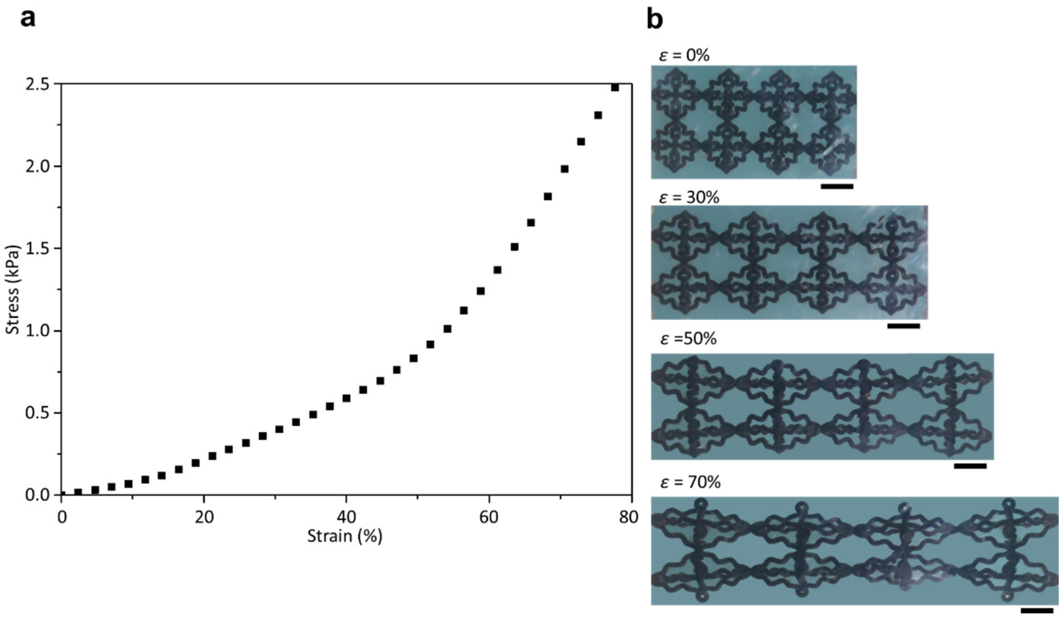

Supplementary Figure 15. Stress-strain curves of a 3D network with the base material made from a mixture of TangoBlackPlus (60%) and VeroWhite (40%), available through the PolyJet 3D printer (Object 350, Stratasys, MN, USA). (a) Measured stress-strain curve of the octahedral network sample (with d_0 = 1.63 mm, p_0 / R_0 = 3, d_0 / R_0 = 1.2, N_0 = 1 and p_j / R_0 = 4). (b) Optical images of the octahedral network structure under different levels of stretching. Scale bar, 10 mm.

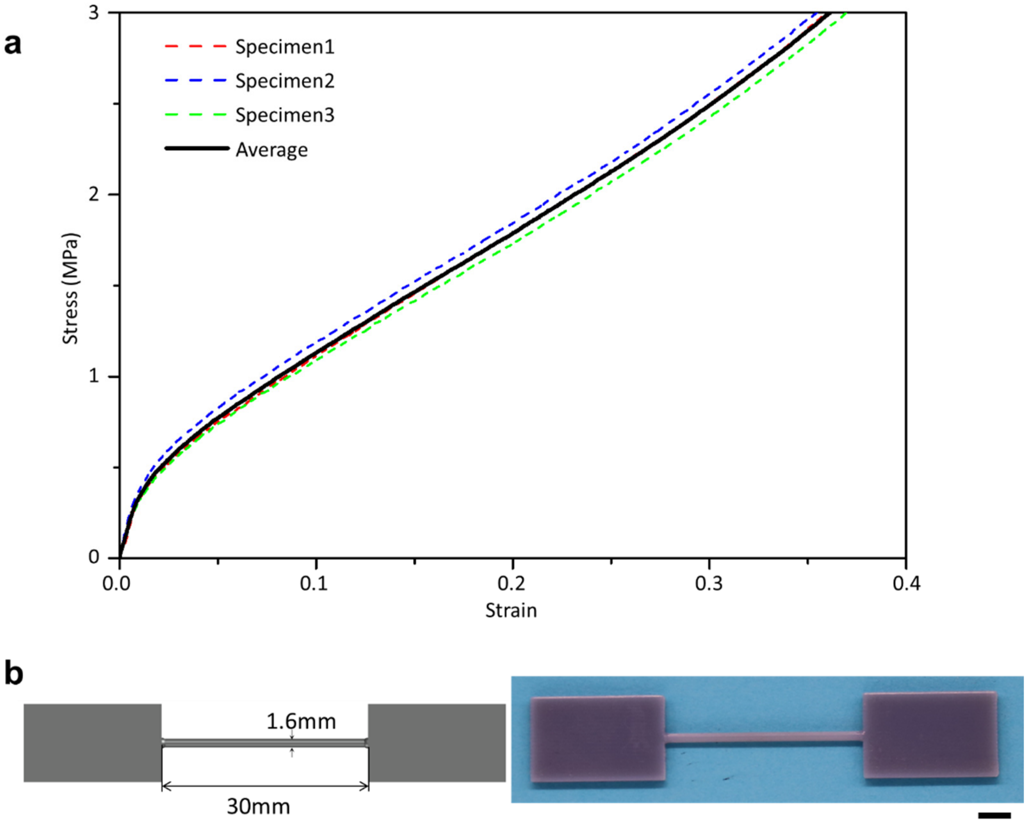

Supplementary Figure 16. The stress-strain curves of the VeroBlue material used in the 3D printing. (a) The experimental stress-strain curves of the VeroBlue material are measured at a low loading rate (0.1 mm·min^{-1}) under the water at a fixed temperature of 25 °C. (b) The Geometric model and optical image of the specimen.

Supplementary Note 1: Geometric parametric equation of the central line associated with 3D helical microstructure.

$$x(\theta)=\begin{cases} R_0\sin^2\dfrac{\pi z^2(\theta)}{2p_\mathrm{j}^2}\sin\theta & 0\le\theta<\dfrac{\pi}{2} \\ R_0\sin\theta & \dfrac{\pi}{2}\le\theta<\left(2N_0+\dfrac{1}{2}\right)\pi \\ R_0\sin^2\dfrac{\pi\left(z(\theta)-N_0p_0-p_\mathrm{j}\right)^2}{2p_\mathrm{j}^2}\sin\theta & \left(2N_0+\dfrac{1}{2}\right)\pi\le\theta<(2N_0+1)\pi \end{cases}$$

$$y(\theta)=\begin{cases} R_0\sin^2\dfrac{\pi z^2(\theta)}{2p_\mathrm{j}^2}\cos\theta & 0\le\theta<\dfrac{\pi}{2} \\ -R_0\cos\theta & \dfrac{\pi}{2}\le\theta<\left(2N_0+\dfrac{1}{2}\right)\pi \\ -R_0\sin^2\dfrac{\pi\left(z(\theta)-N_0p_0-p_\mathrm{j}\right)^2}{2p_\mathrm{j}^2}\cos\theta & \left(2N_0+\dfrac{1}{2}\right)\pi\le\theta<(2N_0+1)\pi \end{cases}$$

$$z(\theta)=\begin{cases} \dfrac{p_0}{4\pi^3}\left(3-\dfrac{16p_\mathrm{j}}{p_0}\right)\theta^3+\dfrac{p_0}{4\pi^2}\theta^2+\dfrac{p_0}{16\pi}\left(\dfrac{48p_\mathrm{j}}{p_0}-5\right)\theta & 0\le\theta<\dfrac{\pi}{2} \\ \dfrac{p_0}{2\pi}\left(\theta-\dfrac{\pi}{2}\right)+p_\mathrm{j} & \dfrac{\pi}{2}\le\theta<\left(2N_0+\dfrac{1}{2}\right)\pi \\ N_0p_0+2p_\mathrm{j}-\dfrac{p_0}{4\pi^3}\left(3-\dfrac{16p_\mathrm{j}}{p_0}\right)\left[(2N_0+1)\pi-\theta\right]^3 & \left(2N_0+\dfrac{1}{2}\right)\pi\le\theta<(2N_0+1)\pi \\ -\dfrac{p_0}{4\pi^2}\left[(2N_0+1)\pi-\theta\right]^2-\dfrac{p_0}{16\pi}\left(\dfrac{48p_\mathrm{j}}{p_0}-5\right)\left[(2N_0+1)\pi-\theta\right] & \end{cases} \tag{1}$$

where d_0, R_0, N_0, p_0, and p_j denote the diameter of the fiber, the radius of the helix, the number of the coil, the pitch, and the joint length, respectively. The total arc length of each joint can be derived as

$$l_\mathrm{j}=\int_0^{\frac{\pi}{2}}\sqrt{\left(\frac{\mathrm{d}x}{\mathrm{d}\theta}\right)^2+\left(\frac{\mathrm{d}y}{\mathrm{d}\theta}\right)^2+\left(\frac{\mathrm{d}z}{\mathrm{d}\theta}\right)^2}\,\mathrm{d}\theta \tag{2}$$

Supplementary Note 2: Design procedure of soft 3D network materials to achieve desired stress-strain curves of biological tissues.

Taking the soft octahedral network materials as an example, an iterative process that uses FEA as a design tool determines the geometric parameters of microstructures for reproducing the stress-strain curves of biological tissues. Firstly, a rough range of the critical strain can be determined according to the target J-shaped stress-strain curve. For a given critical strain, a set of geometric parameters, including the normalized pitch (p_0 / R_0) and the number (N_0) of coil, can be then determined (Fig. 3d). Note that the joint length (p_j / R_0) of the connection segments mainly ensures that the tangling of the microstructures at the nodal regions of the network is avoided, and is not a primary design parameter for the desired mechanical properties. Then, according to the critical stress of the target J-shaped stress-strain curve, the normalized fiber diameter (d_0 / R_0) is determined for each set of p_0 / R_0 and N_0, yielding a group of optimized parameters. Finally, by minimizing the overall difference of the calculated stress-strain curve and the target one, an optimal set of design parameters (d_0 / R_0, p_0 / R_0 and N_0) can be selected from the above group of optimized parameters.
