# Supplementary material for: Soft three-dimensional network materials with rational bio-mimetic designs
Source: Nat Commun. 2020 Mar 4;11:1180. doi: 10.1038/s41467-020-14996-5 (PMC7055264; doi:10.1038/s41467-020-14996-5)
Supplement: Supplementary file 2 — Description of Additional Supplementary Files [file 41467_2020_14996_MOESM2_ESM.pdf]

Description of Additional Supplementary Files

File Name: Supplementary Movie 1

Description: Soft octahedral network material under large levels of deformations (stretching, compression, bending and twisting).
